# Exercise during hemodialysis does not affect the phenotype or prothrombotic nature of microparticles but alters their proinflammatory function

**DOI:** 10.14814/phy2.13825

**Published:** 2018-10-07

**Authors:** Naomi Martin, Alice C. Smith, Maurice R. Dungey, Hannah M. L. Young, James O. Burton, Nicolette C. Bishop

**Affiliations:** ^1^ National Centre for Sport and Exercise Medicine School of Sport, Exercise and Health Sciences Loughborough University Leicestershire United Kingdom; ^2^ Leicester Kidney Lifestyle Team Department of Infection, Immunity & Inflammation University of Leicester and John Walls Renal Unit University Hospitals of Leicester NHS Trust Leicestershire United Kingdom

**Keywords:** Cardiovascular disease, hemodialysis, inflammation, kidney, microparticles

## Abstract

Hemodialysis patients have dysfunctional immune systems, chronic inflammation and comorbidity‐associated risks of cardiovascular disease (CVD) and infection. Microparticles are biologically active nanovesicles shed from activated endothelial cells, immune cells, and platelets; they are elevated in hemodialysis patients and are associated with chronic inflammation and predictive of CVD mortality in this group. Exercise is advocated in hemodialysis to improve cardiovascular health yet acute exercise induces an increase in circulating microparticles in healthy populations. Therefore, this study aimed to assess acute effect of intradialytic exercise (IDE) on microparticle number and phenotype, and their ability to induce endothelial cell reactive oxygen species (ROS) in vitro. Eleven patients were studied during a routine hemodialysis session and one where they exercised in a randomized cross‐over design. Microparticle number increased during hemodialysis (2064–7071 microparticles/*μ*L, *P* < 0.001) as did phosphatidylserine+ (*P* < 0.05), platelet‐derived (*P* < 0.01) and percentage procoagulant neutrophil‐derived microparticles (*P* < 0.05), but this was not affected by IDE. However, microparticles collected immediately and 60 min after IDE (but not later) induced greater ROS generation from cultured endothelial cells (*P* < 0.05), suggesting a transient proinflammatory event. In summary IDE does not further increase prothrombotic microparticle numbers that occurs during hemodialysis. However, given acute proinflammatory responses to exercise stimulate an adaptation toward a circulating anti‐inflammatory environment, microparticle‐induced transient increases of endothelial cell ROS in vitro with IDE may indicate the potential for a longer‐term anti‐inflammatory adaptive effect. These findings provide a crucial evidence base for future studies of microparticles responses to IDE in view of the exceptionally high risk of CVD in these patients.

## Introduction

Chronic kidney disease (CKD) affects approximately 10% of the world's population (Eckardt et al. 2013). Patients with end‐stage renal disease have a dysfunctional immune system that is paradoxically chronically activated and anergic (Betjes 2013); this manifests as systemic inflammation associated with an increased risk of atherosclerosis, cardiovascular disease (CVD), and cachexia (Stenvinkel 2010; Heine et al. 2012). End‐stage renal disease patients are also immunologically vulnerable (Kurts et al. 2013) and have higher risk and greater severity of infections and associated tissue injury (Dalrymple and Go 2008). As such, the progression of CKD is in part mediated by the immune system (Kurts et al. 2013). Hemodialysis (HD) itself is associated with further increased mortality, aggravated by cardiac injury induced by intradialytic hypotension (Shoji et al. 2004), myocardial stunning (Burton et al. 2009), immune activation aggravated by endotoxin influx (Kurts et al. 2013) and biomaterial‐induced complement activation (Nilsson et al. 2007).

Patients with kidney disease have an altered immune and inflammatory profile, possibly due to the uremic environment (Girndt et al. 2001), with increased proinflammatory T‐cells, monocytes, monocyte and neutrophil Toll‐Like Receptor 4 activity and activated platelet aggregates (Ashman et al. 2003; Gollapudi et al. 2010; Kurts et al. 2013). This is associated with elevated concentrations of circulating proinflammatory cytokines including TNF*α*, IL‐6 and the acute phase reactant, C‐reactive protein (Girndt et al. 1995; Costa et al. 2008). Chronic inflammation increases incrementally with CKD progression (Kaysen 2014) and is intrinsically linked to the high risk of mortality and morbidity from CVD in these patients (Stenvinkel 2010). In CKD, microparticles (MP) have proinflammatory actions (Puddu et al. 2010) and have been associated with endothelial damage, increased risk for calcification, anemia, and thrombosis (Gao et al. 2015; Erdbrügger and Li 2016), with endothelial derived MP collected 72 h after last dialysis reported to independently predict all‐cause and CVD mortality in stable HD patients (Amabile et al. 2012). MP are shed from activated or apoptotic sources including immune cells platelets and endothelial cells (Boulanger et al. 2007), with their actions depending on their cellular origin (Barteneva et al. 2013). These intact circulating membrane nanovesicles range in size from 100 to 1000 nm and are composed of plasma membrane and the cytoplasmic contents of their parent cell (Morel et al. 2011). MP act as systemic signaling vehicles and their inflammatory actions include inducing leukocyte aggregation (Forlow et al. 2000), chemotaxis (Barry et al. 1998) and the release of cytokines (Puddu et al. 2010). Indeed, MP can transport and transfer proteins such as tissue factor, a major initiator of blood coagulation, contributing to endothelial dysfunction (Scholz et al. 2002). The actions of MP therefore represent a connection between chronic inflammation and CVD in CKD.

Lower levels of physical activity are a risk factor for CVD yet within the HD population patients are frequently inactive (Johansen et al. 2000) and this is associated with a risk mortality of similar magnitude to that of other well‐established risk factors, such as a one‐point reduction in serum albumin concentration (O'Hare et al. 2003). A recent Cochrane review reported a plethora of benefits of regular exercise for this population, including improvements to cardiovascular health (Heiwe and Jacobson 2014). The so‐called “anti‐inflammatory” effect of regular exercise is thought to play a key role through various different mechanisms that centre on a chronic adaptive response to repeated transient proinflammatory events during and following acute exercise sessions (Gleeson et al. 2011). These include reducing visceral adipose tissue mass and associated inflammatory cytokine release, stimulating transient acute proinflammatory cytokine release from muscle, leading to a counteractive, longer lasting release of anti‐inflammatory mediators and movement from a proinflammatory toward an anti‐inflammatory profile of immune cell phenotypes (Gleeson et al. 2011). Indeed, we have recently reported favorable changes in inflammatory leukocyte phenotype with a reduction in monocytes with a proinflammatory phenotype in patients after 6 months of moderate intensity IDE compared with non‐exercising HD patients (Dungey et al. 2017). This is arguably as an adaptive response to regular exercise given our earlier observation of acute elevations in proinflammatory monocyte phenotypes in response to a single IDE session of the same intensity (Dungey et al. 2015). Acute exercise is also known to induce an increase in circulating MP (Frühbeis et al. 2015) and in healthy populations, transient increases in, platelet‐, monocyte‐, and EC‐derived MP (Lansford et al. 2016) have been reported following acute moderate‐vigorous exercise. These acute elevations are thought to be necessary to stimulate an anti‐inflammatory adaptation with repeated exercise sessions as lower numbers of EC MP are reported in healthy individuals in response to long‐term aerobic training (Kim et al. 2015). Therefore, the aim of this study was to assess the acute effect of IDE on the total number, cellular origin, prothrombotic phenotype and ROS inducing ability of circulating MP. We hypothesized that an acute bout of IDE would induce a greater MP response compared to routine HD.

## Materials and Methods

This novel investigation used samples collected from a subgroup of patients recruited to our previously published study (Dungey et al. 2015). While the trial protocol is the same, the analyses and data presented here are novel and have not been previously published.

### Patients

Eleven patients were recruited from a satellite unit of University Hospitals of Leicester NHS Trust. In this randomized crossover study, patients were studied during a routine HD session and also one where they completed exercise. Exclusion criteria were: age < 18 years, lower limb vascular access, cardiovascular event in the last 3 months, severe heart failure, severe chronic obstructive pulmonary disease, acute liver disease, uncontrolled diabetes mellitus, severe lower limb orthopedic problems, severe lower limb neuromuscular disease, clinically overt infection in the last 6 weeks, pregnancy, insufficient command of English to understand the patient information sheet and give informed consent. All patients had one or more significant non‐renal comorbidity reported in their medical history. Specifically, 2 of the 11 patients had diabetes mellitus (18%), 9 were diagnosed with hypertension (81%), and 5 were diagnosed with heart disease (54%). All patients used polysulfone high‐flux dialyzers. The dialysate, dialyzer, needle size and dialysis duration and prescriptions were unchanged between study days. The study received approval from the NHS Research Ethics Committee (ref. 10/H0406/36), and all patients gave written informed consent to participate. Patient characteristics are represented in Table 1.

**Table 1:** Patients' characteristics

Age (y)	59 ± 10
Sex (*n*)	
Male/Female	7/4
Ethnicity (*n*)	
White British	4 (36%)
Indian	7 (64%)
Height (cm)	166 ± 9
Dry weight (kg)	80.2 ± 19.2
BMI (kg/m^2^)	28.8 ± 6.8
Hemodialysis vintage (y)	3.62 (0.94–3.82)
Access	
AVF	9 (82%)
Catheter	2 (12%)
Primary disease (*n*)	Glomerulonephritis (3)
	Cystic/Poly (2)
	Diabetes (1)
	Pyelonephritis (1)
	Uncertain (3)
	Other (1)
Number of co‐morbidities	4 ± 2 (range 1–7)
Number of medications	12 ± 4 (range 4–16)
aSystolic blood pressure (mmHg)	122 (111–145)
aDiastolic blood pressure (mmHg)	64 (56–71)

Data are mean ± standard deviation, median (interquartile range), or *n* (%).

a Blood pressure variables are based on a 3‐day average taken before (and not including) the first trial day.

### Exercise session

The exercise session has been described in detail previously (Dungey et al. 2015). Briefly, patients participated in 2 trial arms, separated by a week and carried out on the same day each week. For the exercise arm, 60 min into their HD treatment patients performed a 5 min warm‐up followed by a 30 min bout of IDE using a specially designed cycle ergometer (Letto series; Reck, Germany). Patients cycled at self‐selected gears at a rating of perceived exertion of “somewhat hard” (Borg 1973). Patients routinely took part in an exercise program for at least 3 months prior to this study therefore the study here represents the usual effects of regular IDE for these patients. During the control non‐exercising arm of the study patients rested throughout HD.

### Phlebotomy and microparticle isolation

To investigate the acute effects of exercise, blood samples were taken from the HD lines pre‐exercise (60 min of HD), immediately after exercise (post‐exercise, 100 min of HD), 1 h post‐exercise (160 min of HD, and at the end of HD (240 min of HD). In the non‐exercising control sessions bloods were taken at equivalent times of HD. Blood was drawn using a dry syringe and immediately aliquoted into a trisodium citrate pretreated monovette (Sarstedt AG, Nümbrecht, Germany). Platelet‐poor plasma was prepared from blood samples following two centrifugation steps of 2500*g* for 20 min at 4°C, and then stored in 250 *μ*L aliquots at −80°C until use. MP in cell‐free plasma were prepared according to Jy et al. (2004).

### Flow cytometry

**Figure 1:**
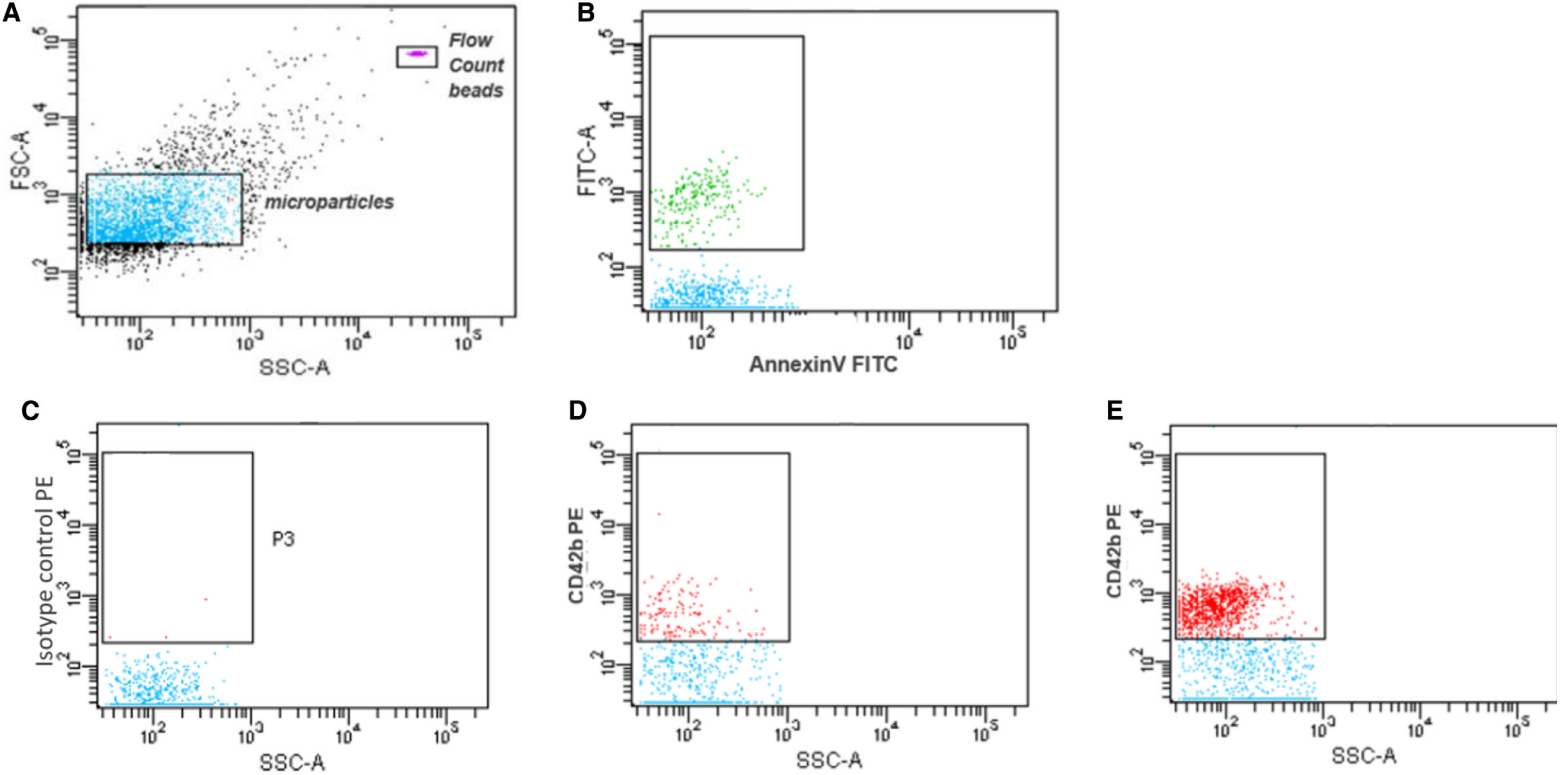
Examples of flow cytometric analysis of microparticles. Representative graphs from cytofluorometric analysis of MP in platelet‐free plasma from non‐exercising CKD patient undergoing HD. (A) Construction of MP gating region using Megamix submicrometer beads (based on (Puddu et al. 2010)) and MP enumeration using Flow‐Count beads. (B) Determination of phosphatidylserine/AnnexinV^+^ MP. Representative phenotyping flow cytometric analysis of MP, comparing positively stained gated MP were determined by comparison with isotype control staining (C). CD42b^+^ (platelet‐derived) MP events from samples taken 60 min into HD (D) to those the end of the HD session (E).

Annexin V (AnV) expression was used to detect phosphatidylserine expression on MP, considered to be prothrombotic (Owens and Mackman 2011). Aliquots of MP were incubated alone, with Annexin V‐fluorescein isothiocyanate (AnV‐FITC) (BD Biosciences, Oxford, UK) as a marker of phosphatidylserine expression, and buffer A (10 mmol/L HEPES, 140 mmol/L NaCl, 2.5 mmol/L CaCl_2_, pH 7.4) or with AnV‐FITC and a calcium‐free control buffer B (10 mmol/L HEPES, 140 mmol/L NaCl, pH 7.4) in the dark for 25 min at RT. Buffer A was added to MP alone, or AnV‐stained MP, while control AnV staining was assessed with by adding buffer B to AnV‐stained MP. Phenotypic labeling was performed with the following mouse anti‐human phycoerythrin(PE)‐labeled antibodies to specific cell surface markers: platelet CD42b, endothelial cell CD144, tissue factor CD142, neutrophil CD66b, monocyte CD14, or appropriate isotype control antibodies (all BD Biosciences, Oxford, UK). MP were also dual‐labeled with phenotyping antibodies as above plus AnV‐FITC. All antibody incubations were carried out as described above and MP were resuspended in buffer A. MP were enumerated using Flow‐Count Fluorospheres™ (Beckman Coulter, High Wycombe, UK). All flow cytometric acquisition was done using a FACSCanto II and all analysis was carried out using FACSDiva™ software (both BD Biosciences, Oxford, UK). MP were acquired using a gating defined by size‐calibrated fluorescent Megamix™ beads according to the manufacturer's instructions (BioCytex, Marseille, France) and according to a standardized calibrated‐bead strategy (Robert et al. 2008). Examples of flow cytometric profiles are shown in Figure 1. All samples were acquired using the slowest flow rate for a fixed time period of 120 seconds.

### MP‐induced reactive oxygen species generation

EA.hy926 EC lines were maintained according to the manufacturer's instructions; they were cultured in DMEM supplemented with 10% (v/v) heat‐inactivated fetal calf serum, 2 mmol/L l‐glutamine, 100 U/mL penicillin, 100 *μ*g/mL streptomycin (all Life Technologies, Warrington, UK) and maintained at 37°C in a humidified 5% CO_2_ atmosphere. EA.hy926 cells were seeded onto black 96‐well plates (Perkin Elmer, Seer Green, UK) at a density of 2.5 × 10^4^ cells/well. The following day the medium was removed and cells were loaded with 5 *μ*mol/L dichloro‐dihydro‐fluorescein diacetate (DCFH‐DA) (Sigma, Gillingham, UK) at a final concentration of 10 *μ*g/mL in medium for 15 min at 37°C in the dark before the addition of 25 *μ*L MP (prepared and enumerated as above), control buffers or medium (Sigma, Gillingham, UK). Fluorescence was measured at 529 nm immediately, every 15 min for 2 h at 37°C and finally after a 20 h incubation at 37°C in a humidified 5% CO_2_ atmosphere, using a Varioskan™ Flash Multimode Reader (Thermo Fisher Scientific, Loughborough, UK). Each treatment was carried out in duplicate.

### Statistics

Treatment conditions and baseline differences between arms were compared using paired *t*‐tests or Wilcoxon signed‐ranks tests where applicable. Two‐factor repeated measures ANOVA was used to analyze data: trial arm (exercise vs. control) × time. Where data were non‐normally distributed (Shapiro‐Wilk) ANOVA was performed on the logarithmic transformation of the data and reported in original form. If the omnibus test found a significant effect post hoc paired *t*‐tests and repeated contrasts were used and adjusted for multiple comparisons using the Holm‐Bonferroni method (Holm 1979). Statistical analysis was performed on Statistical Package for Social Sciences (SPSS v.22, IBM, New York). Data are presented as mean ± standard deviation or median (interquartile range) as described. Statistical significance was accepted at *P* < 0.05.

## Results

### Exercise and hemodialysis

All 11 recruited patients successfully completed 30 min of cycling starting 60 min into HD on the exercise trial. The mean power output was measured as 22.5 ± 7.3 W. Pre HD body mass, ultrafiltration goal and pump speed did not differ between exercise and resting trials (Table 2). No adverse events were reported.

**Table 2:** Hemodialysis treatment variables on exercise and control trial arms

	Exercise	Control	*P* value
Pre‐HD weight (kg)	81.4 ± 19.2	81.5 ± 19.5	0.736
Ultrafiltration goal (L)	1.62 ± 0.63	1.56 ± 0.74	0.545
Pump speed (mL/min)	324 ± 45	324 ± 45	1.000

### Evaluation of the effects of hemodialysis and IDE on total and prothrombotic microparticles

**Figure 2:**
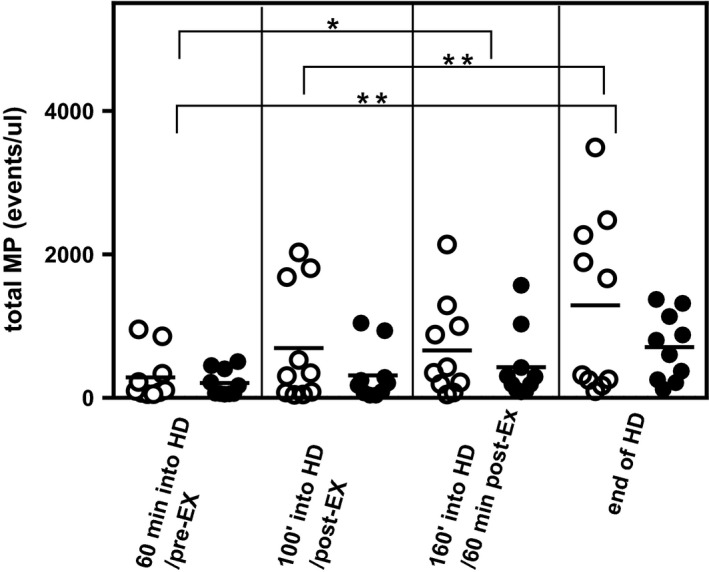
Total numbers of microparticles over the course of HD in exercising and non‐exercising CKD patients. Patients on HD rested (open symbols) or exercised (closed symbols). MP were isolated, then analyzed and enumerated using flow cytometry, from samples taken 60 min into HD, immediately following exercise (or equivalent time in control group), 60 min after exercise (equivalent to 160 min into dialysis for control group) and at the end of HD. Total numbers of MP had increased significantly by the end of HD compared to both 60 min and 100 min (main effect for HD, **P < 0.01 for both) into HD. The numbers of MP at 160 min into HD were also significantly greater than those observed 60 min into HD (*P < 0.05). All of these observed increases were irrespective of IDE. All *n* = 10.

**Figure 3:**
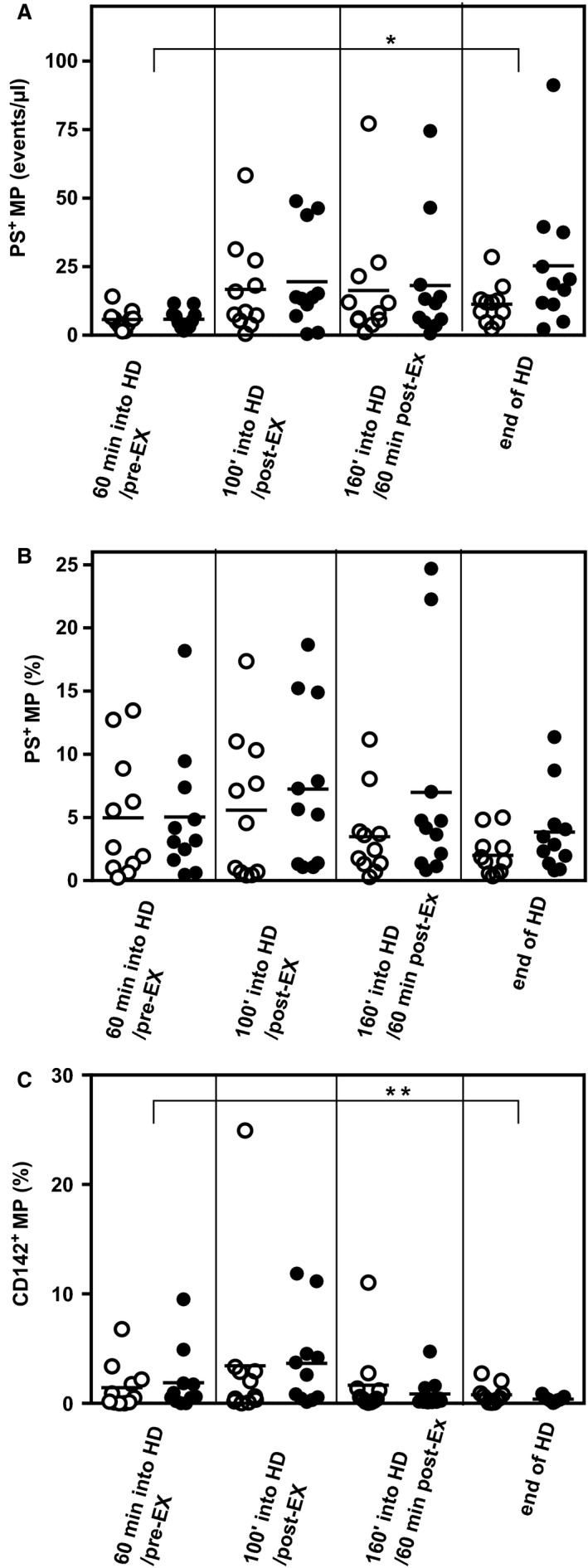
Procoagulant microparticles over the course of HD in exercising and non‐exercising CKD patients. Patients on HD rested (open symbols) or exercised (closed symbols). MP isolated from samples were taken 60 min into HD, immediately following exercise (or equivalent time in control group), 60 min after exercise (equivalent to 160 min into dialysis for control group) and at the end of HD were labeled with AnnexinV which binds to phosphatidylserine, or anti‐CD142, and analyzed using flow cytometry. There was a significant increase in the number of phosphatidylserine‐positive MP (A) in both groups from 60 min into HD compared to at the end of HD (main effect of HD, *P < 0.05), but there was no significant change in the percentage of MP stained with AnV (B) over the course of HD or any differences between exercising or non‐exercising groups. Number of CD142^+^ tissue factor expressing MP were unaffected by HD or IDE (data not shown) yet there was a significant decrease in the percentage of CD142^+^ MP (C) between 100 and 160 min of HD (main effect for HD, *P < 0.05), irrespective of exercise. All *n* = 11.

We observed a significant increase in the number of MP over the course of HD (main effect of HD, *P* < 0.001) (Fig. 2) with a ~4 fold increase from 60 min into HD compared to the end of HD. There was no effect of IDE. The overall number (Fig. 3A), but not % (Fig. 3B), of MP expressing phosphatidylserine increased significantly over the course of HD (main effect of HD, *P* < 0.05), with increases seen in 22 out of the 26 HD sessions observed. Over the same time there was a decrease (*P *=* *0.066) in % (but not number) of circulating MP expressing tissue factor (Fig. 3C). Again IDE had no effect on these responses.

### Evaluation of the effects of hemodialysis and IDE on the cellular origin of microparticles

**Figure 4:**
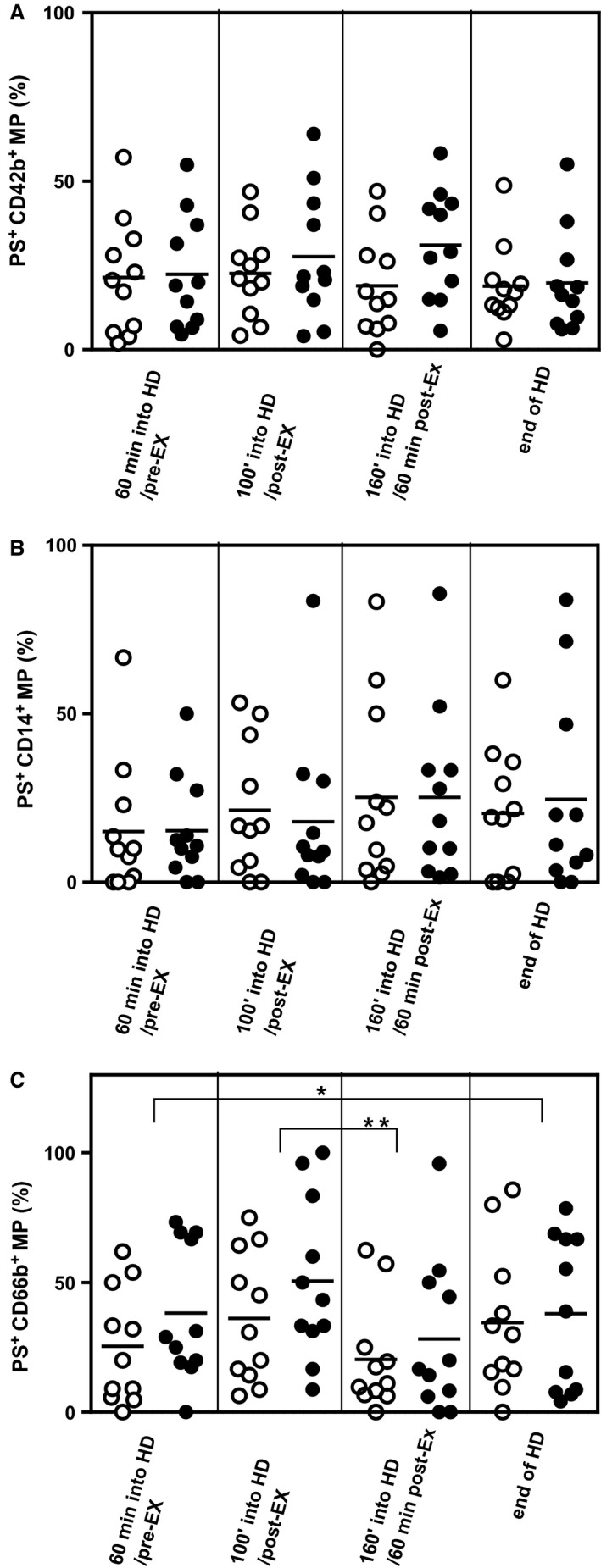
Procoagulant phenotypic microparticles over the course of HD in exercising and non‐exercising CKD patients. Patients on HD rested (open symbols) or exercised (closed symbols). MP isolated from platelet‐free plasma samples were taken 60 min into HD, immediately following exercise (or equivalent time in control group), 60 min after exercise (equivalent to 160 min into dialysis for control group) and at the end of HD and stained with AnnexinV‐FITC and PE‐conjugated phenotyping antibodies to determine presence of phosphatidylserine (PS) and cellular origin, and analyzed using flow cytometry. Procoagulant PS +  MP derived from platelets (CD42b+) (A) and monocytes (CD14 + ) (B) were unaffected by HD or IDE. The % PS+ neutrophil‐derived MP (C) decreased 1.8‐fold between 100 min (triangles; 43.3 ± 28.4%) and 160 min into HD (squares; 24.2 ± 25.4%) (*P < 0.05). IDE had no effect on these responses. All *n* = 11.

There was a significant increase in the number of platelet‐derived CD42b+MP by the end of HD (main effect of HD, *P* < 0.001, Table 3). There were no other significant changes in the numbers of MP derived from the other cellular origins studied, but there was a trend (main effect of HD, *P *=* *0.08) for an increase in monocyte‐derived CD14 + MP over the course of HD (Table 3). IDE had no effect on these responses. The % procoagulant phosphatidylserine+ MP derived from platelets (Fig. 4A), EC (data not shown) and monocytes (Fig. 4B) were unaffected by HD or exercise. However, % neutrophil‐derived phosphatidylserine+CD66b+MP decreased between 100 and 160 min of HD (43.3 ± 28.4% to 24.2 ± 25.4%), main effect of HD, *P* < 0.05; Fig. 4C). Again IDE had no effect on this response.

**Table 3:** Effects of hemodialysis and intradialytic exercise on numbers of circulating microparticles derived from platelets, endothelial cells, neutrophils, and monocytes

MP phenotype (MP/*μ*L)	60 min of HD pre‐ex	100 min into HD post‐ex	160 min into HD 60 min post‐ex	End of HD
Platelet^a^				
Rest	8.82 ± 7.12	20.2 ± 17.0	15.6 ± 15.8	30.3 ± 18.2
Exercise	11.0 ± 7.40	19.2 ± 21.4	15.6 ± 13.8	27.8 ± 31.5
Endothelial cell				
Rest	0.21 ± 0.44	0.30 ± 0.30	0.08 ± 0.16	0.16 ± 0.54
Exercise	0.20 ± 0.40	0.08 ± 0.28	0.25 ± 0.57	0.00 ± 0.00
Neutrophil				
Rest	6.51 ± 8.52	6.74 ± 5.40	6.25 ± 7.15	7.18 ± 6.04
Exercise	2.84 ± 3.22	6.09 ± 6.53	3.57 ± 4.42	5.93 ± 7.37
Monocyte				
Rest	4.05 ± 5.23	7.71 ± 10.38	4.54 ± 2.62	12.62 ± 11.51
Exercise	4.08 ± 4.20	5.58 ± 3.93	5.11 ± 4.18	5.18 ± 3.03

All data are expressed as mean ± SD MP/*μ*L, from 11 patients studied.

a *P* < 0.001 platelet‐derived CD42b+MP 60 min into HD versus end of HD; (main effect of HD only).

### Evaluation of ROS‐inducing effects of microparticles from dialyzing and intradialytic exercising patients

**Figure 5:**
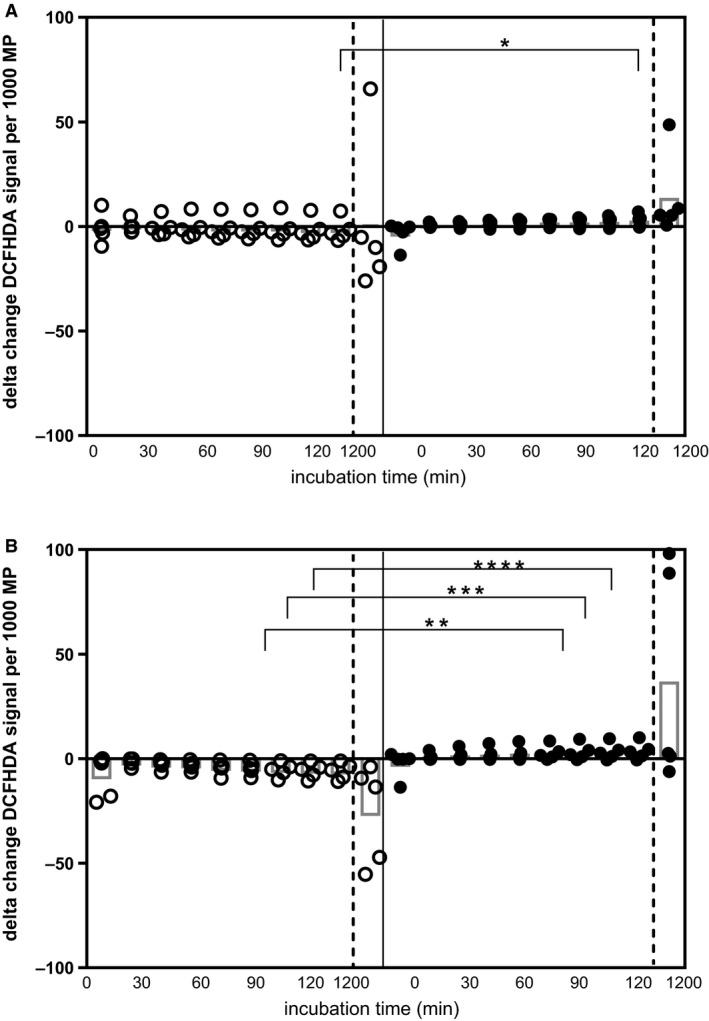
Microparticle induced reactive oxygen species in an endothelial cell line. MP isolated from plasma samples were taken 60 min into HD, immediately following exercise (open symbols) (or equivalent time in control group (closed symbols)) (A) and 60 min after exercise (equivalent to 160 min into dialysis for control group) (B) and were incubated with EA.hy926 endothelial cells (EC) which had been preloaded with 5 *μ*mol/L DCFHDA. Reactive oxygen species (ROS) generation was analyzed every 15 min for 120 min and then after 20 h using a fluorescent plate reader. All ROS responses were adjusted to signal per 1000 MP following MP enumeration as described and delta changes were calculated relative to the sample taken 60 min into HD. MP from exercising patients, taken immediately after exercise induced significantly increased ROS when incubated with EC for 120 min (*P < 0.05) compared to MP collected from these patients at the same time at rest (A). Also, MP taken from exercising patients 60 min after exercise induced significantly increased levels of ROS when incubated with EC at various time points (90 min: **, 105 min: ***, 120 min: ****, all *P* < 0.05) compared to the response induced by MP from patients at rest 160 min into HD (B). All *n* = 5.

The ROS‐inducing capabilities of MP taken from patients while exercising and at rest during HD were investigated to provide insight into their function and potential involvement in pathophysiology. MP collected from exercising patients immediately after their IDE session (Fig. 5A) had significantly greater ability to induce ROS after 120 min incubation with EC compared to MP collected at the same time (100 min into HD) when resting (delta change of 2.82 ± 3.03 compared to −1.68 ± 5.42 DCFHDA fluorescence units per 1000 MP; *P* < 0.05). This increased ROS induction in EC was still evident in MP from patients 60 min after their exercise session (Fig. 5B) compared to MP taken at the same time at rest (delta change of 3.72 ± 4.04 compared to −5.99 ± 3.94 DCFHDA fluorescence units per 1000 MP; *P* < 0.05). There were no differences between trial arms at the end of HD.

## Discussion

This is the first study to comprehensively characterize the acute response of circulating MP to IDE during HD and to investigate the ability of these circulating MP to induce EC ROS in vitro. Our findings suggest that IDE at an intensity that can be sustained for 30 min does not exacerbate the effects of HD in increasing the number and phenotype of prothrombotic circulating MP. However, MP collected after IDE induced greater ROS production from cultured EC, suggesting an acute, short‐lived proinflammatory response; this could indicate the potential for chronic anti‐inflammatory adaptation with regular IDE. Given the association between MP, inflammation and endothelial dysfunction in CKD (Erdbrügger and Li 2016), the link between MP and cardiovascular mortality (Amabile et al. 2012) in HD patients, and the known anti‐inflammatory effects of exercise (Gleeson et al. 2011) these findings provide new insight into the effect of the HD process on cardiovascular risk in end stage renal disease and the impact of IDE on this risk.

To the best of our knowledge, there are no other studies to date reporting enumeration of, or changes in, the total number of circulating MP during HD. Previous work by our group looking at total numbers of circulating MP in HD patients did not observe numbers during the HD process itself (Burton et al. 2013). In addition, this study used Nanoparticle‐tracking analysis to enumerate MP without any surface marker detection. We used flow cytometry with MP fluorescent intensity using size‐calibrated fluorescent beads is as a threshold for particle detection. This approach offers better separation from background noise, is less likely affected by aggregation of particles and might detect particles as low as 100–200 nm (Erdbrügger and Li 2016) and although one technique was used here to enumerate and analyze MP, recent publications assert that flow cytometry remains the most suitable method for determining the cellular origin on MP (Maas et al. 2015; Erdbrügger and Li 2016; Cointe et al. 2017). It was not possible for us to phenotype MP in this study using Western blotting due to the small sample volumes obtained (Pospichalova et al. 2015). A recent study highlighting the limitations of both Nanosight particle tracking analysis and tunable resistive pulse sensing (Maas et al. 2015) suggests that flow cytometry is the most sensitive and accurate method for enumeration of MP. Therefore, flow cytometry is the most appropriate method for the phenotypic enumeration analyses required to address the hypothesis of this study, and no other single method would provide such comprehensive information.

Contact of blood with foreign materials and vascular shear stress during HD causes activation of immune cells, the complement system, cytokine production and release and oxidative stress (Amore and Coppo 2002) despite the dilution of uremic toxins. Subjecting blood to conditions simulating flow itself induces increases in MP numbers in vitro (Holtom et al. 2011), while adding uremic plasma (Dursun et al. 2009) or uremic toxins (Faure et al. 2006) to cultured EC cell lines induces endothelial dysfunction and MP release in vitro. Thus, it is of no surprise that we observed an acute increase in total MP numbers, although exercise performance had no effect, possibly due to the relatively low workload that patients were able to perform here. This is in line with our previous findings that the IDE used here has little impact on HD‐induced neutrophil activation and inflammatory cytokine release (Dungey et al. 2015). Other studies investigating the effects of exercise on MP have studied healthy younger subjects and used moderate‐vigorous incremental exercise protocols (Chaar et al. 2011; Wahl et al. 2014; Frühbeis et al. 2015) with exercise beginning at ~100 W. This far exceeds the capabilities of the patients studied here (average 22 W). Therefore, although the protocol used here did not induce many exercise‐related changes in *numbers* of MP, ours was a pragmatic approach geared toward assessing the responses to the exercise capabilities of our patient population.

Changes in circulating prothrombotic MP were observed over the course of HD, but again no differences were detected between the exercising or resting study arms. The number of phosphatidylserine +MP increased over the course of HD yet the number of MP expressing tissue factor did not change, although there was a decrease in the proportion of these. Although phosphatidylserine and tissue factor are both considered prothrombotic molecules, recent research suggests that they have different roles in the coagulation process (Spronk et al. 2014), with phosphatidylserine considered a potent inducer of cell‐surface tissue factor conversion into its prothrombotic decrypted state (Rao et al. 2012). Therefore, the increase in phosphatidylserine+ MP may have subsequent prothrombotic impacts beyond the observation period of this study.

The overall percentage of phosphatidylserine+MP remained unchanged both over the course of HD and during each arm of the study but, at an average of 4% phosphatidylserine+MP of the total MP population, was considerably lower than the 90% that has been previously reported in HD patients when not on dialysis (Trappenburg et al. 2012). Our values may reflect clearance during HD, but perhaps more likely to reflect methodological and definition variations known to cause discrepancies in MP numbers and indeed phosphatidylserine expression in earlier studies (Ayers et al. 2011; Erdbrügger and Li 2016). Values of around 11% phosphatidylserine+MP have been reported in healthy males (Nielsen et al. 2014). AnV is commonly used, as in this study, to detect surface expression of its phospholipid ligand, phosphatidylserine but it is not clear how much PS is externalized on MP and not all MP bind AnV (Erdbrügger and Li 2016). AnV and phosphatidylserine expression alone should not be used as a definitive marker for all MP, but should be applied to consider populations of both phosphatidylserine+ and phosphatidylserine− MP. Without consistency in definitions comparisons between studies remain difficult.

Increased numbers of MP derived from platelets and EC are known to be associated with risk factors for CVD with EC‐derived MP predictive of all cause and CVD mortality in stable HD patients (Amabile et al. 2012). We found an increase in platelet‐derived CD42b+MP over the course of HD, irrespective of whether exercise had taken place or not. However, we found no changes in phosphatidylserine+CD42b+MP. Platelet‐derived CD41 + MP (Daniel et al. 2006; Faure et al. 2006) and CD42 + MP (Burton et al. 2013) have been shown to be increased in number in both nondialysis CKD and HD patients compared to healthy controls, and differences may be again related to the use of phosphatidylserine expression to define MP (Erdbrügger and Li 2016).

We detected extremely low, or absent, circulating EC‐derived CD144 + MP in patients when resting or exercising during HD. Other investigations report decreases in CD41‐CD31 + MP (Boulanger and Dignat‐George 2011) and CD144 + MP (Trappenburg et al. 2012), or no changes in either CD144 + MP or CD146 + MP (Faure et al. 2006) following an HD session. CD144 and CD105 are the only constitutive markers of EC which are not found on other cell types (Latham et al. 2015) and although a variety of endothelial markers are used by groups studying EC‐derived MP, the dynamics, stimuli and mechanisms of EC MP formation are little understood (Curtis et al. 2013). It is thought that EC‐derived MP account for a very minor proportion of all circulating MP (Trappenburg et al. 2012); indeed, they have been described as “rare” (Tushuizen et al. 2011). While it might be expected that these MP would increase in number during HD because of shear stresses and endothelial activation, the decrease or absence found here and by other groups may be due to the restoration of viscosity and laminar shear stress during HD (Amabile et al. 2005) as high‐flux filtration has been shown to reduce the number of EC‐derived MP (Ramirez et al. 2007).

MP effectively act as a storage pool of bioactive effectors (Hugel et al. 2005) and are known to induce EC ROS (Holtom et al. 2011). Here, we were able to detect ROS produced when EC were incubated with MP collected up to 60 min after exercise, but not with MP derived from the end of HD or from any MP samples taken when these patients were resting during HD. While endothelial ROS act as important second messengers within the cells, an imbalance between ROS generation and innate antioxidant defences represents the primary cause of endothelial dysfunction and the initiation of vascular diseases (Chen et al. 2018). The consequences of sustained increases in EC ROS production include the recruitment of inflammatory cells, proinflammatory cytokine release, reduced nitric oxide vasodilatory activity, enhanced formation of the deleterious radical, peroxynitrite and cell death (El Assar et al. 2013; Barhoumi et al. 2014). However, here the effect was short‐lived (<2 h). Acute, transient proinflammatory events lasting only a few hours are a key stimulus for longer‐term adaptations to regular exercise that are associated with reduced CVD risk (Gleeson et al. 2011). In support of this, mouse EC subjected to laminar shear stress exhibited enhanced NADPH oxidase subunit expression which was reduced after 2 weeks of moderate intensity aerobic training (Robinson et al. 2017) and EPO‐enhanced ROS generation and monocyte/macrophage infiltration were alleviated after 8 weeks of moderate intensity aerobic exercise in mice with endothelin‐1 overexpression (Barhoumi et al. 2014). Furthermore, enhanced in vivo NADPH oxidase‐derived ROS production from EC in the muscle microvasculature was attenuated after 8 weeks of moderate intensity aerobic training in sedentary obese populations (La Favor et al. 2016). The transient, yet enhanced, ROS production from EC cultured with post‐exercise MP could reflect an initial stimulus for this positive adaptive process but it must be acknowledged that the small sample size does not allow definitive conclusions to be made.

Given the pragmatic approach of this study some limitations must be acknowledged. The randomized‐crossover design allows direct comparisons to be made between the two trial arms and reduces the potential impact of confounding elements such as circadian rhythm. The sample size precludes definitive conclusions from being made but does provide crucial data to inform the design of future investigations. Patients eligible to take part in exercise were healthier with a lower risk of complications and while the first sample was taken before exercise, this was 60 min into HD. This was to allow for patient stabilization and to avoid interference of routine HD set‐up, but it is certainly possible that increases in MP occurred in that first hour. However, the purpose of this study was to determine the effects of an acute session of IDE on MP responses, not the effects of HD per se. Furthermore, although patients involved in this study had been taking part in the IDE program for at least 3 months, this was not a longitudinal study. The longer‐term effects of IDE on MP responses are still unknown. However, a recent study in renal transplant recipients reported no changes in circulating phosphatidylserine+MP after 6 months of aerobic training yet observed a significant increase in prothrombotic MP in non‐exercising recipients, perhaps indicating an adaptive amelioration of vascular activation in the exercising group (Pitha et al. 2015).

In summary, given the known inflammatory properties of MP these novel findings indicate that an acute session of moderate intensity IDE does not add further to the heavy inflammatory burden of HD, extending our previous findings (Dungey et al. 2015). However, acute proinflammatory responses to exercise are an avenue for the development of a longer term anti‐inflammatory adaptation (Gleeson et al. 2011). The ability of MP collected immediately and 60 min after exercise (but not later than this) to induce a transient amplification of EC ROS production in vitro may indicate the potential for a longer‐term positive adaptive effect of regular IDE on endothelial cell function. Given the exceptionally high risk of CVD in these patients, these novel findings provide an evidence‐base for future study of the long‐term effects of regular IDE on MP phenotype and prothrombotic properties.

## Conflict of Interest

Outside the submitted work Reck UK funded MRD, HMLY, and JOB to attend the 2012 BMJ Awards. There are no other financial conflicts of interest. The results presented in this paper have not been published previously in whole or part, except in abstract format, at the 2016 European Renal Association‐European Dialysis and Transplant Association Meeting.

## References

[phy213825-bib-0001] Amabile, N. , A. P. Guérin , A. Leroyer , Z. Mallat , C. Nguyen , J. Boddaert , et al. 2005 Circulating endothelial microparticles are associated with vascular dysfunction in patients with end‐stage renal failure. J. Am. Soc. Nephrol. 16:3381–3388. 16192427 10.1681/ASN.2005050535

[phy213825-bib-0002] Amabile, N. , A. P. Guérin , A. Tedgui , C. M. Boulanger , and G. M. London . 2012 Predictive value of circulating endothelial microparticles for cardiovascular mortality in end‐stage renal failure: a pilot study. Nephrol. Dial. Transplant. 27:1873–1880. 22036944 10.1093/ndt/gfr573

[phy213825-bib-0003] Amore, A. , and R. Coppo . 2002 Immunological basis of inflammation in dialysis. Nephrol. Dial. Transplant. 17:16–24. 10.1093/ndt/17.suppl_8.16 12147772

[phy213825-bib-0004] Ashman, N. , M. G. Macey , S. L. Fan , U. Azam , and M. M. Yaqoob . 2003 Increased platelet‐monocyte aggregates and cardiovascular disease in end‐stage renal failure patients. Nephrol. Dial. Transplant. 18:2088–2096. 13679485 10.1093/ndt/gfg348

[phy213825-bib-0005] Ayers, L. , M. Kohler , P. Harrison , I. Sargent , R. Dragovic , M. Schaap , et al. 2011 Measurement of circulating cell‐derived microparticles by flow‐cytometry: sources of variability within the assay. Thromb. Res. 127:370–377. 21257195 10.1016/j.thromres.2010.12.014

[phy213825-bib-0006] Barhoumi, T. , M. Briet , D. A. Kasal , J. C. Fraulob‐Aquino , N. Idris‐Khodja , P. Laurant , et al. 2014 Erythropoietin‐induced hypertension and vascular injury in mice overexpressing human endothelin‐1: exercise attenuated hypertension, oxidative stress, inflammation and immune response. J. Hypertens. 32:784–794. 24463938 10.1097/HJH.0000000000000101

[phy213825-bib-0007] Barry, O. P. , D. Pratico , R. C. Savani , and G. A. Fitzgerald . 1998 Modulation of monocyte–endothelial cell interactions by platelet microparticles. J. Clin. Invest. 102:136–144. 9649567 10.1172/JCI2592 PMC509075

[phy213825-bib-0008] Barteneva, N. S. , E. Fasler‐Kan , M. Bernimoulin , J. N. H. Stern , E. D. Ponomarev , L. Duckett , et al. 2013 Circulating microparticles: square the circle. BMC Cell Biol. 14:23–44. 23607880 10.1186/1471-2121-14-23 PMC3651414

[phy213825-bib-0009] Betjes, M. 2013 Immune cell dysfunction and inflammation in end‐stage renal disease. Nat. Rev. Nephrol. 9:255–265. 23507826 10.1038/nrneph.2013.44

[phy213825-bib-0010] Borg, G. A. V. 1973 Perceived exertion: a note on “history” and methods. Med. Sci. Sports Exerc. 5:90–93. 4721012

[phy213825-bib-0011] Boulanger, C. M. , and F. Dignat‐George . 2011 Microparticles: an introduction. Arterioscler. Thromb. Vasc. Biol. 31:2–3. 21160061 10.1161/ATVBAHA.110.220095

[phy213825-bib-0012] Boulanger, C. M. , N. Amabile , A. P. Guérin , B. Pannier , A. S. Leroyer , C. N. Mallat , et al. 2007 In vivo shear stress determines circulating levels of endothelial microparticles in end‐stage renal disease. Hypertension 9:902–908. 10.1161/01.HYP.0000259667.22309.df 17309952

[phy213825-bib-0013] Burton, J. O. , H. J. Jefferies , N. M. Selby , and C. W. McIntyre . 2009 Hemodialysis‐induced cardiac injury: determinants and associated outcomes. Clin. J. Am. Soc. Nephrol. 4:914–920. 19357245 10.2215/CJN.03900808 PMC2676185

[phy213825-bib-0014] Burton, J. O. , H. A. Hamali , R. Singh , N. Abbasian , R. Parsons , A. K. Patel , et al. 2013 Elevated levels of procoagulant plasma microvesicles in dialysis patients. PLoS ONE 8:e72663. 23936542 10.1371/journal.pone.0072663 PMC3732282

[phy213825-bib-0015] Chaar, V. , M. Romana , J. Tripette , C. Broquere , M. G. Huisse , O. Hue , et al. 2011 Effect of strenuous physical exercise on circulating cell‐derived microparticles. Clin. Hemorheol. Microcirc. 47:15–25. 21321404 10.3233/CH-2010-1361

[phy213825-bib-0016] Chen, Q. , Q. Wang , J. Zhu , Q. Xiao , and L. Zhang . 2018 Reactive oxygen species: key regulators in vascular health and diseases. Br. J. Pharmacol. 175:1279–1292. 10.1111/bph.13828 28430357 PMC5867026

[phy213825-bib-0017] Cointe, S. , C. Judicone , S. Robert , M. J. Mooberry , P. Poncelet , M. Wauben , et al. 2017 Standardization of microparticle enumeration across different flow cytometry platforms: results of a multicentre collaborative workshop. J. Thromb. Haemost. 15:187–193. 27662257 10.1111/jth.13514 PMC5280151

[phy213825-bib-0018] Costa, E. , M. Lima , J. M. Alves , S. Rocha , P. Rocha‐Pereira , E. Castro , et al. 2008 Inflammation, T‐Cell phenotype, and inflammatory cytokines in chronic kidney disease patients under hemodialysis and its relationship to resistance to recombinant human erythropoietin therapy. J. Clin. Immunol. 28:268–275. 18205031 10.1007/s10875-007-9168-x

[phy213825-bib-0019] Curtis, A. M. , J. Edelberg , R. Jonas , W. T. Rogers , J. S. Moore , W. Syed , et al. 2013 Endothelial microparticles: sophisticated vesicles modulating vascular function. Vasc. Med. 18:204–214. 23892447 10.1177/1358863X13499773 PMC4437568

[phy213825-bib-0020] Dalrymple, L. S. , and A. S. Go . 2008 Epidemiology of acute infections among patients with chronic kidney disease. Clin. J. Am. Soc. Nephrol. 3:1487–1493. 18650409 10.2215/CJN.01290308 PMC4571152

[phy213825-bib-0021] Daniel, L. , F. Fakhouri , D. Joly , L. Mouthon , P. Nusbaum , J. P. Grunfeld , et al. 2006 Increase in circulating neutrophil and platelet microparticles during acute vasculitis and hemodialysis. Kidney Int. 69:1416–1423. 16531979 10.1038/sj.ki.5000306

[phy213825-bib-0022] Dungey, M. , N. C. Bishop , H. M. L. Young , J. O. Burton , and A. C. Smith . 2015 The impact of exercising during haemodialysis on blood pressure, markers of cardiac injury and systemic inflammation – preliminary results of a pilot study. Kidney Blood Press. Res. 40:593–604. 26619202 10.1159/000368535

[phy213825-bib-0023] Dungey, M. , H. M. L. Young , D. R. Churchward , J. O. Burton , A. C. Smith , and N. C. Bishop . 2017 Regular exercise during haemodialysis promotes an anti‐inflammatory leucocyte profile. Clin Kidney J 10:813–821. 29225811 10.1093/ckj/sfx015 PMC5716206

[phy213825-bib-0024] Dursun, I. , H. M. Poyrazoglu , Z. Gunduz , H. Ulger , A. Yykylmaz , R. Dusunsel , et al. 2009 The relationship between circulating endothelial microparticles and arterial stiffness and atherosclerosis in children with chronic kidney disease. Nephrol. Dial. Transplant. 24:2511–2518. 19244228 10.1093/ndt/gfp066

[phy213825-bib-0025] Eckardt, K. U. , J. Coresh , O. Devuyst , R. J. Johnson , A. Köttgen , A. S. Levey , et al. 2013 Evolving importance of kidney disease: from subspeciality to global health burden. Lancet 382:158–169. 23727165 10.1016/S0140-6736(13)60439-0

[phy213825-bib-0026] El Assar, M. , J. Angulo , and L. Rodríguez‐Mañas . 2013 Oxidative stress and vascular inflammation in aging. Free Radic. Biol. Med. 65:380–401. 23851032 10.1016/j.freeradbiomed.2013.07.003

[phy213825-bib-0027] Erdbrügger, U. , and T. H. Li . 2016 Extracellular vesicles in renal diseases: more than novel biomarkers? J. Am. Soc. Nephrol. 27:12–26. 26251351 10.1681/ASN.2015010074 PMC4696584

[phy213825-bib-0028] Faure, V. , L. Dou , F. Sabatier , C. Cerini , J. Sampol , Y. Berland , et al. 2006 Elevation of circulating endothelial microparticles in patients with chronic renal failure. J. Thromb. Haemost. 4:566–573. 16405517 10.1111/j.1538-7836.2005.01780.x

[phy213825-bib-0029] Forlow, S. B. , R. P. McEver , and M. U. Nollert . 2000 Leukocyte‐leukocyte interactions mediated by platelet microparticles under flow. Blood 95:1317–1323. 10666205

[phy213825-bib-0030] Frühbeis, C. , S. Helmig , S. Tug , P. Simon , and E. M. Krämer‐Albers . 2015 Physical exercise induces rapid release of small extracellular vesicles into the circulation. J. Extracell Vesicles 4:28239. 26142461 10.3402/jev.v4.28239 PMC4491306

[phy213825-bib-0031] Gao, C. , R. Xie , C. Yu , R. Ma , Y. Dong , H. Meng , et al. 2015 Thrombotic role of blood and endothelial cells in uremia through phosphatidylserine exposure and microparticle release. PLoS ONE 10:1–16. 10.1371/journal.pone.0142835 PMC4646287 26580207

[phy213825-bib-0032] Girndt, M. , H. Kohler , E. Schiedhelm‐Weick , J. F. Schlaak , K. H. Meyer zum Buschenfelde , and B. Fleischer . 1995 Production of interleukin‐6, tumor necrosis factor alpha and interleukin‐10 in vitro correlates with the clinical immune defect in chronic hemodialysis patients. Kidney Int. 47:559–565. 7723241 10.1038/ki.1995.70

[phy213825-bib-0033] Girndt, M. , M. Sester , U. Sester , H. Kaul , and H. Köhler . 2001 Molecular aspects of T‐ and B‐cell function in uremia. Kidney Int. 78:S206–S211. 10.1046/j.1523-1755.2001.59780206.x 11169012

[phy213825-bib-0034] Gleeson, M. , N. C. Bishop , D. J. Stensel , M. R. Lindley , S. S. Mastana , and M. A. Nimmo . 2011 The anti‐inflammatory effects of exercise: mechanisms and implications for the prevention and treatment of disease. Nat. Rev. Immunol. 11:607–615. 21818123 10.1038/nri3041

[phy213825-bib-0035] Gollapudi, P. , J. W. Yoon , S. Gollapudi , M. V. Pahl , and N. D. Vaziri . 2010 Leukocyte toll‐like receptor expression in end‐stage kidney disease. Am. J. Nephrol. 31:247–254. 20090311 10.1159/000276764

[phy213825-bib-0036] Heine, G. H. , A. Ortiz , Z. A. Massy , B. Lindholm , A. Wiecek , A. Martines‐Castelao , et al. 2012 Monocyte subpopulations and cardiovascular risk in chronic kidney disease. Nat. Rev. Nephrol. 8:362–369. 22410492 10.1038/nrneph.2012.41

[phy213825-bib-0037] Heiwe, S. , and S. H. Jacobson . 2014 Exercise training in adults with CKD: a systematic review and meta‐analysis. Am. J. Kidney Dis. 64:383–393. 24913219 10.1053/j.ajkd.2014.03.020

[phy213825-bib-0038] Holm, S. 1979 Simple sequentially rejective multiple test procedure. Scand. J. Statist. 6:65–70.

[phy213825-bib-0039] Holtom, E. , J. R. Usherwood , M. G. Macey , and C. Lawson . 2011 Microparticle formation after co‐culture of human whole blood and umbilical artery in a novel in vitro model of flow. Cytometry 81A:390–399. 10.1002/cyto.a.22010 22213485

[phy213825-bib-0040] Hugel, B. , M. C. Martinez , C. Kunzelmann , and J. M. Freyssinet . 2005 Membrane microparticles: two sides of the coin. Physiology 20:22–27. 15653836 10.1152/physiol.00029.2004

[phy213825-bib-0041] Johansen, K. L. , G. M. Chertow , A. V. Ng , K. Mulligan , S. Carey , P. Y. Schoenfeld , et al. 2000 Physical activity levels in patients on hemodialysis and healthy sedentary controls. Kidney Int. 57:2564–2570. 10844626 10.1046/j.1523-1755.2000.00116.x

[phy213825-bib-0042] Jy, W. , L. L. Horstman , J. J. Jimenez , Y. S. Ahn , E. Biró , R. Nieuwland , et al. 2004 Measuring circulating cell‐derived microparticles. J. Thromb. Haemost. 2:1842–1851. 15456497 10.1111/j.1538-7836.2004.00936.x

[phy213825-bib-0043] Kaysen, G. A. 2014 Progressive inflammation and wasting in patients with ESRD. Clin. J. Am. Soc. Nephrol. 9:225–226. 24458072 10.2215/CJN.12541213 PMC3913250

[phy213825-bib-0044] Kim, J. S. , B. Kim , H. Lee , S. Thakkar , D. M. Babbitt , S. Eguchi , et al. 2015 Shear stress‐induced mitochondrial biogenesis decreases the release of microparticles from endothelial cells. Am. J. Physiol. Heart Circ. Physiol. 309:H425–H433. 26024684 10.1152/ajpheart.00438.2014 PMC4525091

[phy213825-bib-0045] Kurts, C. , U. Panzer , H. J. Anders , and A. J. Rees . 2013 The immune system and kidney disease: basic concepts and clinical implications. Nat. Rev. Nephrol. 13:738–753. 10.1038/nri3523 24037418

[phy213825-bib-0046] La Favor, J. D. , G. S. Dubis , H. Yan , J. D. White , M. A. Nelson , E. J. Anderson , et al. 2016 Microvascular endothelial dysfunction in sedentary, obese humans is mediated by NADPH oxidase: influence of exercise training. Arterioscler. Thromb. Vasc. Biol. 36:2412–2420. 27765769 10.1161/ATVBAHA.116.308339 PMC5123754

[phy213825-bib-0047] Lansford, K. A. , D. D. Shill , A. B. Dicks , M. P. Marshburn , W. M. Southern , and N. T. Jenkins . 2016 Effect of acute exercise on circulating angiogenic cell and microparticle populations. Exp. Physiol. 101:155–167. 26487283 10.1113/EP085505

[phy213825-bib-0048] Latham, S. L. , N. Tiberti , N. Gokoolparsadh , K. Holdaway , P. O. Couraud , G. E. Grau , et al. 2015 Immuno‐analysis of microparticles: probing at the limits of detection. Sci. Rep. 5:16314. 26553743 10.1038/srep16314 PMC4639787

[phy213825-bib-0049] Maas, S. L. N. , J. de Vrij , E. J. van der Vlist , B. Geragousian , L. van Bloois , E. Mastrobattista , et al. 2015 Possibilities and limitations of current technologies for quantification of biological extracellular vesicles and synthetic mimics. J. Contr. Rel. 200:87–96. 10.1016/j.jconrel.2014.12.041 PMC4324667 25555362

[phy213825-bib-0050] Morel, O. , L. Jesel , J. M. Freyssinet , and F. Toti . 2011 Cellular mechanisms underlying the formation of circulating microparticles. Arterioscler. Thromb. Vasc. Biol. 31:15–26. 21160064 10.1161/ATVBAHA.109.200956

[phy213825-bib-0051] Nielsen, M. H. , H. Beck‐Nielsen , M. N. Andersen , and A. Handberg . 2014 A flow cytometric method for characterisation of circulating cell‐derived microparticles in plasma. J. Extracell. Vesicles 3:20795. 10.3402/jev.v3.20795 PMC3916676 24511371

[phy213825-bib-0052] Nilsson, B. , K. Nilsson Ekdahl , T. E. Mollnes , and J. D. Lambris . 2007 The role of complement in biomaterial‐induced inflammation. Mol. Immunol. 44:82–94. 16905192 10.1016/j.molimm.2006.06.020

[phy213825-bib-0053] O'Hare, A. M. , K. Tawney , P. Bacchetti , and K. L. Johansen . 2003 Decreased survival among sedentary patients undergoing dialysis: results from the dialysis morbidity and mortality study wave 2. Am. J. Kidney Dis. 41:447–454. 12552509 10.1053/ajkd.2003.50055

[phy213825-bib-0054] Owens, A. P. , N., Mackman . 2011 Microparticles in hemostasis and thrombosis. Circ. Res. 108:1284–1297. 21566224 10.1161/CIRCRESAHA.110.233056 PMC3144708

[phy213825-bib-0055] Pitha, J. , I. Králova Lesná , P. Stávek , A. Mahrová , J. Racek , A. Sekerková , et al. 2015 Effect of exercise on markers of vascular health in renal transplant recipients. Physiol. Res. 64:945–949. 26447524 10.33549/physiolres.933123

[phy213825-bib-0056] Pospichalova, V. , J. Svoboda , Z. Dave , A. Kotrbova , K. Kaiser , D. Klemova , et al. 2015 Simplified protocol for flow cytometry analysis of fluorescently labelled exosomes and microvesicles using dedicated flow cytometer. J. Extracell. Ves. 4:1. 10.3402/jev.v4.25530 PMC4382613 25833224

[phy213825-bib-0057] Puddu, P. , G. M. Puddu , E. Cravero , S. Muscari , and A. Muscari . 2010 The involvement of circulating microparticles in inflammation, coagulation and cardiovascular diseases. Can. J. Cardiol. 26:e140–e145. 10.1016/s0828-282x(10)70371-8 PMC2886541 20386775

[phy213825-bib-0058] Ramirez, R. , J. Carracedo , A. Merino , S. Nogueras , M. A. Alvarez‐Lara , M. Rodríguez , et al. 2007 Microinflammation induces endothelial damage in hemodialysis patients: the role of convective transport. Kidney Int. 72:108–113. 17429343 10.1038/sj.ki.5002250

[phy213825-bib-0059] Rao, L. V. M. , H. Kothari , and U. R. Pendurthi . 2012 Tissue factor: mechanisms of decryption. Front Biosci. 4:1513–1527. 10.2741/477 PMC3883586 22201972

[phy213825-bib-0060] Robert, S. , P. Poncelet , R. Lacroix , L. Arnaud , L. Giraudo , A. Hauchard , et al. 2008 Standardization of platelet‐derived microparticle counting using calibrated beads and a cytomics FC500 routine flow cytometer: a first step towards multicenter studies? J. Thromb. Haemost. 7:190–197. 18983485 10.1111/j.1538-7836.2008.03200.x

[phy213825-bib-0061] Robinson, A. T. , I. S. Fancher , V. Sudhahar , J. T. Bian , M. D. Cook , A. M. Mahmoud , et al. 2017 Short‐term regular aerobic exercise reduces oxidative stress produced by acute in the adipose microvasculature. Am. J. Physiol. Heart Circ. Physiol. 312:H896–H906. 28235790 10.1152/ajpheart.00684.2016 PMC5451589

[phy213825-bib-0062] Scholz, T. , U. Temmler , S. Krause , S. Heptinstall , and W. Losche . 2002 Transfer of tissue factor from platelets to monocytes: role of platelet‐derived microvesicles and CD62P. Thromb. Haemost. 88:1033–1038. 12529756

[phy213825-bib-0063] Shoji, T. , Y. Tsubakihara , M. Fujii , and E. Imai . 2004 Hemodialysis‐associated hypotension as an independent risk factor for two‐year mortality in hemodialysis patients. Kidney Int. 66:1212–1220. 15327420 10.1111/j.1523-1755.2004.00812.x

[phy213825-bib-0064] Spronk, H. M. , H. ten Cate , and P. E. van der Meijden . 2014 Differential roles of tissue factor and phosphatidylserine in activation of coagulation. Thromb. Res. 133:S54–S56. 24759145 10.1016/j.thromres.2014.03.022

[phy213825-bib-0065] Stenvinkel, P. 2010 Chronic kidney disease: a public health priority and harbinger of premature cardiovascular disease. J. Intern. Med. 268:456–467. 20809922 10.1111/j.1365-2796.2010.02269.x

[phy213825-bib-0066] Trappenburg, M. C. , M. van Schilfgaarde , F. C. Frerichs , H. M. Spronk , H. ten Cate , C. W. de Fijter , et al. 2012 Chronic renal failure is accompanied by endothelial activation and a large increase in microparticle numbers with reduced procoagulant capacity. Nephrol. Dial. Transplant. 27:1446–1453. 21873622 10.1093/ndt/gfr474

[phy213825-bib-0067] Tushuizen, M. E. , M. Diamant , A. Sturk , and R. Nieuwland . 2011 Cell‐derived microparticles in the pathogenesis of cardiovascular disease: friend or foe*?* Arterioscler. Thromb. Vasc. Biol. 31:4–9. 21160062 10.1161/ATVBAHA.109.200998

[phy213825-bib-0068] Wahl, P. , F. Jansen , S. Achtzehn , T. Schmitz , W. Bloch , J. Mester , et al. 2014 Effects of high intensity training and high volume training on endothelial microparticles and angiogenic growth factors. PLoS ONE 9:e96024. 24770423 10.1371/journal.pone.0096024 PMC4000202

